# Composite Multivariate Multi-Scale Permutation Entropy and Laplacian Score Based Fault Diagnosis of Rolling Bearing

**DOI:** 10.3390/e24020160

**Published:** 2022-01-21

**Authors:** Wanming Ying, Jinyu Tong, Zhilin Dong, Haiyang Pan, Qingyun Liu, Jinde Zheng

**Affiliations:** 1 School of Mechanical Engineering, Anhui University of Technology, Maanshan 243032, China; wmying033@126.com (W.Y.); pansea@ahut.edu.cn (H.P.); lqyahjx@ahut.edu.cn (Q.L.); jdzheng@ahut.edu.cn (J.Z.); 2 Key Laboratory of Advanced Manufacturing Technology, Beijing University of Technology, Beijing 100124, China; DongZL18133679022@emails.bjut.edu.cn

**Keywords:** rolling bearing, fault diagnosis, multivariate multi-scale permutation entropy, composite multivariate multi-scale permutation entropy, Laplacian score

## Abstract

As a powerful tool for measuring complexity and randomness, multivariate multi-scale permutation entropy (MMPE) has been widely applied to the feature representation and extraction of multi-channel signals. However, MMPE still has some intrinsic shortcomings that exist in the coarse-grained procedure, and it lacks the precise estimation of entropy value. To address these issues, in this paper a novel non-linear dynamic method named composite multivariate multi-scale permutation entropy (CMMPE) is proposed, for optimizing insufficient coarse-grained process in MMPE, and thus to avoid the loss of information. The simulated signals are used to verify the validity of CMMPE by comparing it with the often-used MMPE method. An intelligent fault diagnosis method is then put forward on the basis of CMMPE, Laplacian score (LS), and bat optimization algorithm-based support vector machine (BA-SVM). Finally, the proposed fault diagnosis method is utilized to analyze the test data of rolling bearings and is then compared with the MMPE, multivariate multi-scale multiscale entropy (MMFE), and multi-scale permutation entropy (MPE) based fault diagnosis methods. The results indicate that the proposed fault diagnosis method of rolling bearing can achieve effective identification of fault categories and is superior to comparative methods.

## 1. Introduction

Rolling bearings has an indispensable role in many large rotating machines; once it works with a local failure, the normal operation of mechanical equipment will be disturbed, and serious economic losses and safety incidents will be caused if the fault cannot be detected in time [1,2]. Hence, it is of significance to implement health monitoring and early fault diagnosis of rolling bearing for a safe production [3,4].

The non-stationary and non-linear characteristics often appear at the vibration signals of rolling bearing in actual working conditions [5]. Lots of nonlinear dynamic methods, such as permutation entropy (PE) [6], sample entropy (SE) [7], and fuzzy entropy (FE) [8] were proposed to extract the non-linear dynamic characteristics of time series, which have been widely used in the field of mechanical fault diagnosis. These entropy algorithms above can effectively check the dynamic mutation and randomness of time series, and have the advantage of strong robustness and simple calculation. In Ref. [9], the PE method has been used to extract the fault features by comparing it with the approximate entropy and Lempel-Ziv complexity. In Ref. [10], an approach based on local characteristic-scale decomposition (LCD) and the PE method was put forward for the diagnosis of faulty rolling bearings. In Ref. [11], the SE of the first few components were selected as the characteristic vectors to identify different states of rolling bearings. In Ref. [12], an intelligent fault diagnosis method based on FE and the least square support vector machine (LS-SVM) is proposed. The above entropy-based methods have acquired a good performance on the early fault intelligent diagnosis for rolling bearings. However, they are limited by the single-scale analysis of these entropy methods and tend to ignore the information of time series when processing multi-scale signals. As shown in [13], time-domain features such as mean, root mean square (RMS), standard deviation (SD), and impulse factor, etc., can be successfully used for the condition monitoring of low-speed slew bearing. The advanced statistical based features, such as skewness and kurtosis, can also be applied to the signal, which is non-linear and non-stationary [14]. Ensemble Empirical Mode Decomposition (EEMD) [15] is also a useful method for fault signal analysis. An effective fault diagnosis method based on EEMD and kernel principal component analysis (KPCA) was proposed in [16], where the fault features can be successfully extracted and the fault diagnosis task is well completed. The spectral kurtosis (SK) concept was firstly proposed by Dwyer [17] to achieve more accurate fault feature detection. However, the SK method was not widely used in fault diagnosis field due to its complexity and the loss of definition. To solve this problem, a complete definition of SK was given by Antoni [18], in which the SK is defined as fourth-order spectral cumulant of energy normalization.

To reduce the loss of information, multi-scale entropy (MSE) was introduced in Ref. [19] by analyzing the complex signals from the multi-scale perspective. The methods of multi-scale permutation entropy (MPE) [20], multi-scale fuzzy entropy (MFE) [21], multi-scale dispersion entropy (MDE) [22] have also been proposed in the past few years. In Ref. [23], the EEMD method and MSE were combined to measure the complexity of faulty gearbox signals. In Ref. [24], the approach based on LCD, MPE, and radial basis function network (RBFNs) were proposed and applied to the diagnosis of faulty gear. Although outstanding results can be acquired by the multi-scale methods above, multi-channel signals from different directions from the same location cannot be handled well. To address this shortcoming, a variety of methods for multi-channel signals have been proposed in recent years. In Ref. [25], the multivariate multi-scale sample entropy (MMSE) was proposed and applied into brain consciousness analysis. In Ref. [26], multivariate multi-scale distribution entropy (MMDE) was put forward and used to evaluate the complexity of traffic systems. In Ref. [27], the multivariate multi-scale fuzzy entropy (MMFE) method was introduced and applied to the field of rotating machinery equipment fault diagnosis. Multivariate multi-scale permutation entropy (MMPE) was put forward in Ref. [28], which regarded the multi-channel data as a unique value in the multi-scale way and the MMPE is developed based on the multiple coarse-grained procedures of MPE. Hence, the key drawback of MPE still exists in the MMPE method, namely the lack of precise estimation of feature values.

To overcome the shortcoming of MMPE, an improved method, composite multivariate multi-scale permutation entropy (CMMPE), is proposed in this paper. In the CMMPE method, the composite coarse-grained procedure is used to achieve a more stable and reliable complexity measurement of multivariate time series. Furthermore, the modal recognition is the essence of mechanical fault diagnosis. Hence, an effective recognition algorithm should be performed after the CMMPE calculation. First, to reduce the dimension of CMMPE feature vectors and information redundancy, the Laplacian score (LS) [29] method is used in this paper to reorder the results based on the importance of features, and the LS method has a good performance in the selection of valuable fault features. Next, the bat algorithm optimized support vector machine (BA-SVM) [30] is used to identify the fault types adaptively. The BA-SVM method has the advantages of noise robustness and simple algorithms, and can obtain a higher calculation efficiency, which is beneficial to real-time fault identification. Finally, according to the reliable and stable results of CMMPE, an intelligent fault diagnosis method for rolling bearing is put forward based on the CMMPE, LS, and BA-SVM, where the following three steps are contained. (1) The CMMPE values of vibration signals are computed to extract the hidden fault features. (2) The LS algorithm is used to sort the raw features according to their importance from high to low for sensitive feature selection. (3) The BA-SVM based multi-classifier is built and applied to realize automatic fault recognition. The test data are applied to the CMMPE, LS, and BA-SVM based fault diagnosis methods by comparing the test data with that of MMPE and MPE based methods, respectively. The comparison results indicate that the proposed fault diagnosis approach surpasses the contrasted approaches in terms of recognition rate and fault type diagnosis.

The remaining framework is constructed as follows. The algorithms of MPE and MMPE are reviewed, then the CMMPE method is introduced in Section 2. In Section 3, the stability comparisons between CMMPE and MMPE are performed by simulated signals analysis. In Section 4, the CMMPE, LS, and BA-SVM based fault diagnosis approach is put forward and used in the test data. Finally, the conclusion is drawn in Section 5.

## 2. Introduction of CMMPE Method

In this section, the CMMPE method is developed to promote the insufficient coarse-grained process in MMPE and enhance the ability in handling multi-channel signals. First, the MPE and MMPE methods are reviewed.

### 2.1. The Multi-Scale Permutation Entropy (MPE) Method

To assess randomness and detect dynamic changes in time series, the MPE approach was devised based on PE. The processes of MPE are described as below.

(1) In terms of time series $\left\{x(i){,}_{}i=1{,}_{}2{,}_{}\cdot \cdot \cdot {,}_{}M\right\}$, the coarse-grained time series can be built as

(1) $$y_{j}^{\left(\tau \right)}=\frac{1}{\tau }{\sum_{i=(j-1)\tau +1}^{j\tau }x_{i}}_{{}_{}},\;\;\;1\leq j\leq M/\tau$$

where $\tau_{}(\tau \in N^{*})$ means the scale factor and *j* stands for the length of coarse-grained time series, $y_{j}^{\left(\tau \right)}$ represents the time series $y_{1}^{\tau },y_{2}^{\tau },\cdots ,y_{\tau }^{\tau }$ with a length of p=M/τ, and $y_{j}^{\left(\tau \right)}$ is the same as the raw time series when τ=1.

(2) MPE can be obtained by computing the PE of each coarse-grained time series under every scale factor τ.

(2) $$MPE(x,\tau ,m,t)=PE(y_{j}^{\left(\tau \right)},m,t)$$

where *m* stands for the embedding dimension of PE and *t* means the time delay.

**Figure 1:**
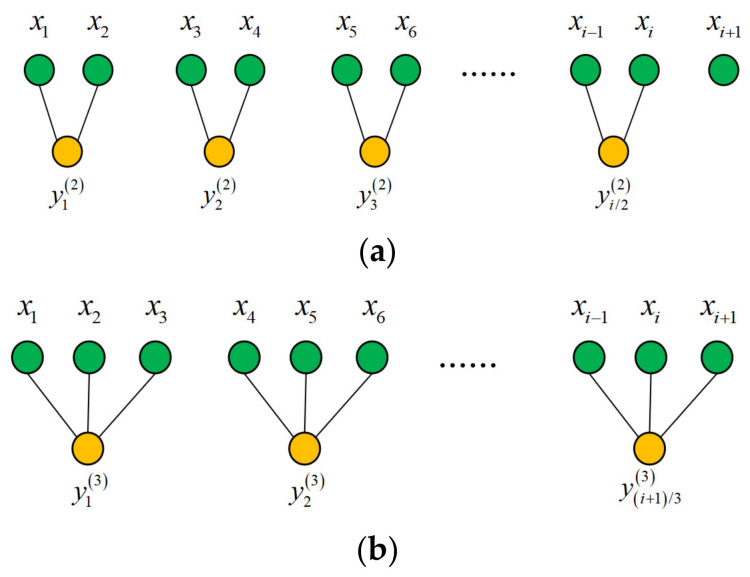
The coarse-grained procedure of MPE. (**a**) τ=2, (**b**) τ=3.

According to the preceding definition of MPE, a higher scale factor indicates a shorter coarse-grained time series with a higher entropy error, which could result in a significant loss of information in the original time series. Moreover, MPE is commonly used to handle the single-channel signal, but it has a weak ability in dealing with the multi-channel data acquired by multi-channel sensors from the same location but in different directions. The coarse-grained process illustration of MPE when τ=2 and τ=3 is shown in Figure 1.

### 2.2. The Multivariate Multi-Scale Permutation Entropy (MMPE) Method

The MMPE method was introduced to promote the capacity of MPE in dealing with multi-channel data. The procedures of MMPE are given as follows.

(1) In terms of multivariate signal ${\left\{x_{k,i}{,}_{}k=1{,}_{}2{,}_{}\ldots {,}_{}M\right\}}_{i=1}^{N}$, which can be recorded as the matrix M×N, where *M* represents the number of signal channels, *N* means the length of time series. For a scale factor τ, the elements of multivariate coarse-grained time series can be indicated as

(3) $$y_{k,j}^{\left(\tau \right)}=\frac{1}{\tau }\sum_{i=(j-1)\tau +1}^{j\tau }x_{k,i},\;\;\;\;1\leq j\leq N/\tau$$

(2) The MMPE value of $y_{k,j}^{\left(\tau \right)}$ can be calculated by

(4) $$MMPE\;\;(x,\tau ,m,t)=PE\;\;(y_{k,j}^{\left(\tau \right)},m,t)\;$$

where *m* stands for the embedding dimension and *t* means the time delay.

It can be seen from Equation (3) that the multi-channel signals are processed based on initial coarse-grained procedure of MPE. Hence, the information loss of original time series still exists in the MMPE method.

### 2.3. The Introduction of the Proposed CMMPE Method

The CMMPE approach is proposed in this subsection to improve the precision of coarse-grained processes in MMPE, and the stages are shown below.

In terms of time series ${\left\{x_{k,i}{,}_{}k=1{,}_{}2{,}_{}\ldots {,}_{}M\right\}}_{i=1}^{N}$, which can be computed to get the composite multivariate coarse-grained time series by

(5) $$y_{k,l,j}^{\left(\tau \right)}=\frac{1}{\tau }{\sum_{i=(j-1)\tau +l}^{j\tau +l-1}x_{k,i}}_{},\;\;\;\;1\leq j\leq N/\tau ,\;\;\;\;1\leq l\leq \tau$$

where $y_{k,l,j}^{\left(\tau \right)}$ stands for *j*-th value of *l*-th coarse-grained time series under *k*-th channel data and with a scale factor of τ.

The PE values of $y_{k,l,j}^{\left(\tau \right)}$ are calculated and CMMPE can be obtained by averaging PE values under the same scale factor.

(6) $$CMMPE(x,\tau ,m,t)=\frac{1}{\tau }\sum_{k=1}^{\tau }PE(y_{k,l,j}^{\left(\tau \right)},m,t)\;\;\;\;\;$$

**Figure 2:**
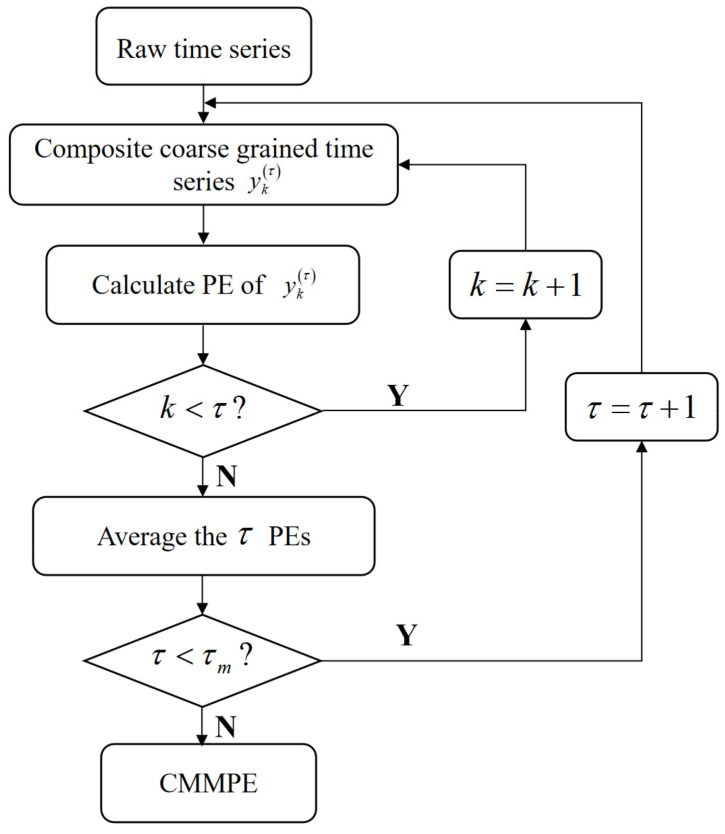
The flowchart of our proposed CMMPE method.

The flowchart of CMMPE is shown in Figure 2.

## 3. The Analysis of Simulated Signal

In this section, three independent and interference-free channels time series generated by white noise and 1/*f* noise are applied to illustrate the effectiveness and superiority of CMMPE in detecting the complexity of multi-channel signals. Several trivariate time series are constructed with a decreasing level of white noise (from 3 to 0) and an increasing level of 1/*f* noise (from 0 to 3). According to Ref. [31], the recommended parameters are preset as follows: the embedding dimension m is set at 6, the length of time series *N* is selected to be 2048, which can satisfy the requirement of *m!* << *N* (means 6*!* << 2048), and the time delay *t* is set as 1. To ensure the validity and stability of results, for both white noise and 1/*f* noise, 20 sets are randomly selected with a length of 2048 and the scale factor τ is set at 30.

**Figure 3:**
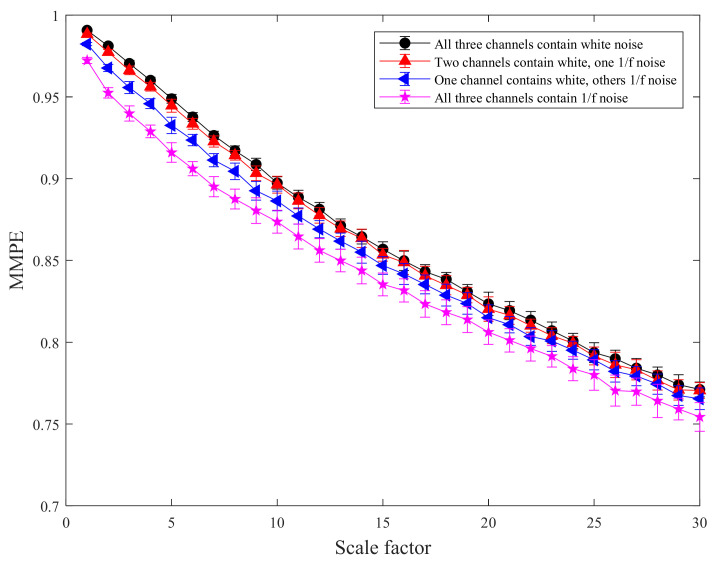
The mean standard deviation curves of MMPE under different scale factors.

**Figure 4:**
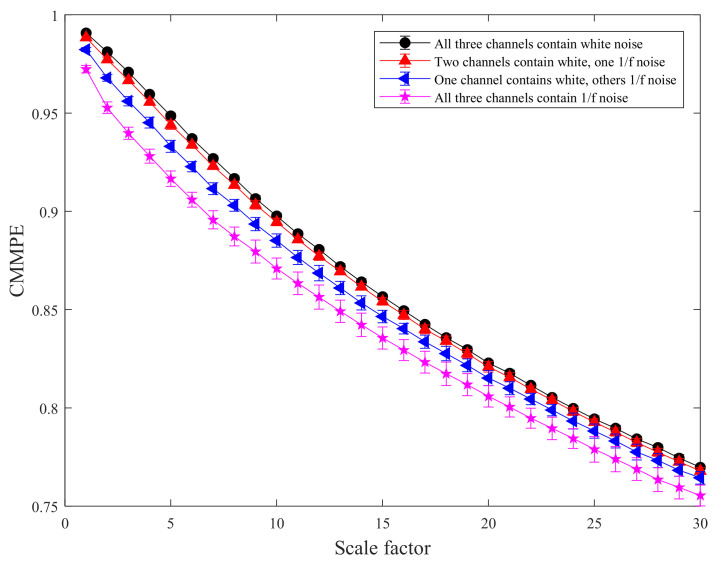
The mean standard deviation curves of CMMPE under different scale factors.

**Figure 5:**
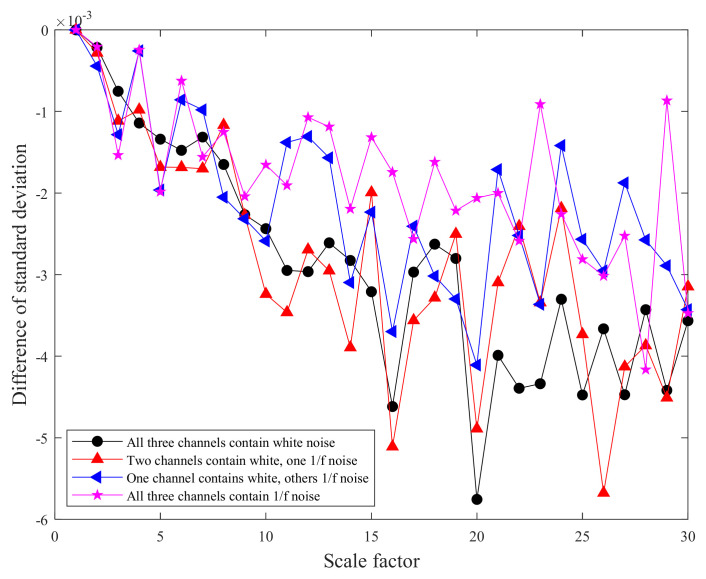
The difference of standard deviation curves between CMMPE and MMPE under different scale factors.

The mean standard deviation curves of MMPE and CMMPE under different scale factors are shown in Figure 3 and Figure 4, respectively. In addition, the mean standard deviation of MMPE and CMMPE decreased with the increasing level of 1/*f* noise, because the arrangement pattern of symbol sequences becomes more regular with the increasing of 1/*f* noise. To quantitatively reflect the difference between CMMPE and MMPE, the mean standard deviation of MMPE is subtracted from that of CMMPE under the same scale and the result is indicated in Figure 5, from which we can see that the mean standard deviation of CMMPE is less than that of MMPE under the same scale factor, which illustrates that CMMPE is more stable than MMPE in feature extraction.

## 4. The CMMPE, LS, and BA-SVM Based Intelligent Fault Diagnosis Method

Modal recognition is the fundamental of mechanical fault diagnosis. As a result, after feature extraction, an effective pattern recognition algorithm should be used to reliably identify the defect types. First, the LS method is used in this paper to reduce the dimensionality and information redundancy of the CMMPE feature vector. Next, the BA-SVM method is used to realize automatic identification of failure modes. The LS, BA-SVM, and the proposed intelligent fault diagnosis methods are introduced below.

### 4.1. Laplacian Score for Feature Selection

The effectiveness and superiority of CMMPE in reflecting the hidden nonlinear dynamic fault features have been proved above. However, information redundancy and low recognition rate can be caused by feature abundance. Hence, an effective optimal feature selection method is necessary for higher calculation accuracy and lower computation complexity. The LS-based feature selection method is introduced to rearrange the features according to their importance from high to low, and sensitive fault features are then randomly selected for training and testing. The complex high-dimensional feature space can be changed into a simple low-dimensional feature space by the LS reordering. The basic idea of LS is to evaluate characteristic value by local preserving ability, and a smaller LS score represents a more important feature value. The overall geometric structure information contained in fault signal feature set can be well retained by the LS method, which is more helpful for fault diagnosis. The effectiveness and superiority of the LS method have been verified in the Ref. [31].

### 4.2. The Bat Optimization Algorithm Based Support Vector Machine

After the raw features are rearranged by LS, an appropriate pattern recognition method should be selected to realize automatic identification of fault categories. The SVM is a commonly used pattern recognition method in fault diagnosis, and its classification accuracy mainly depends on two parameters: penalty factor *C* and kernel function *g*. To obtain the best parameters and optimum classification effect, an intelligent bionic algorithm called BA-SVM is introduced in this paper and the procedures are given as follows. According to Ref. [30], the bat population number is set as 10 and the number of iterations is 150.

### 4.3. The Proposed Fault Diagnosis Method of Rolling Bearing

In this subsection, an intelligent fault diagnosis method for rolling bearing is introduced on the basis of CMMPE, LS and BA-SVM, and the procedures are exhibited as below.

(1) For given *K* categories of rolling bearings (*K* = 13 in this paper), *N* samples are selected for each type, and each sample contains *M*-channel (*M* = 3 in this paper) signals. Each the sample data is then analyzed by CMMPE under a scale factor of *S* (*S* = 30 in this paper), and the CMMPE values will be taken as a representation of sample information to form the original feature sets $M\times R^{N\times S}$.
(2) Type *i* samples from *N* samples are randomly selected as the training feature, noted as $M\times R^{i\times S}$, and then the remaining samples are selected as the testing feature sets, noted as $M\times R^{(N-i)\times S}$.
(3) LS is applied to rearrange the raw training features from low to high on the basis of their LS scores, and the first several sensitive features are selected to rebuild the training feature sets. Accordingly, the testing sets are also rearranged as the sensitive fault testing sets according to the LS scores.
(4) The sensitive sets of training samples are put into the BA-SVM based multi-classifier for training.
(5) The sensitive sets of testing samples are input to the trained multi-classifier to intelligently recognize fault categories according to the outputs.

**Figure 6:**
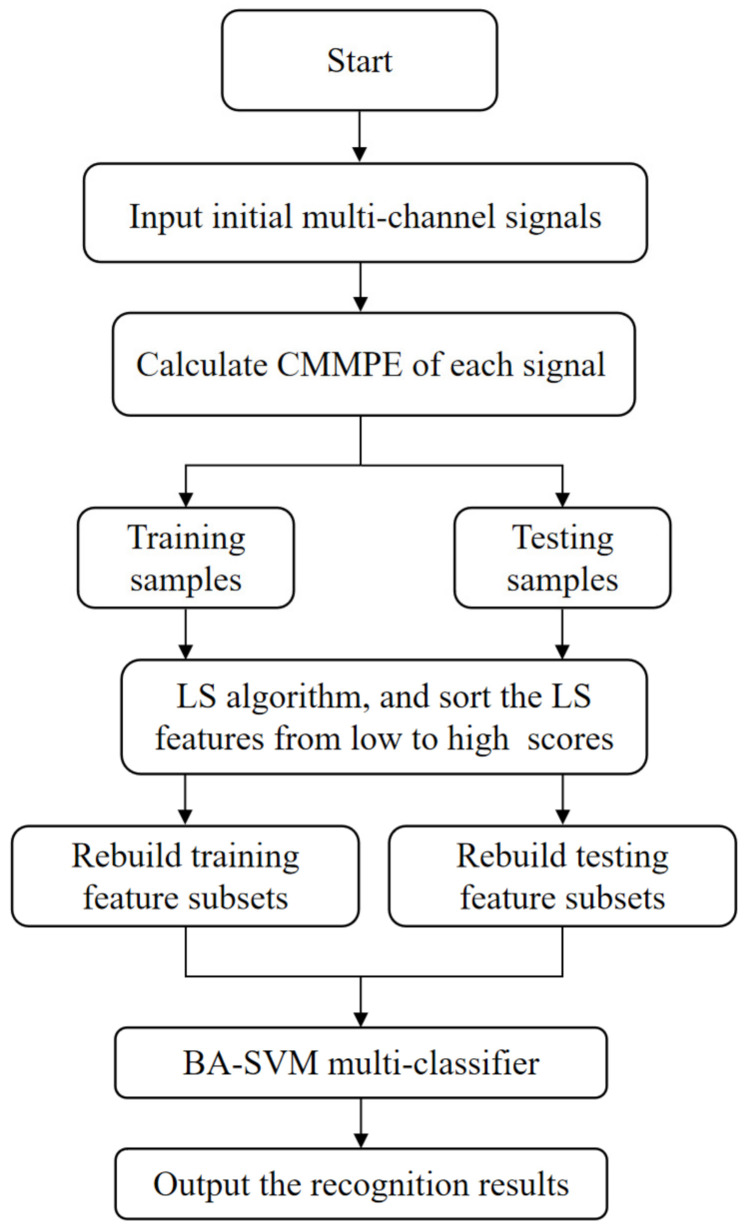
The flowchart of proposed fault diagnosis method.

The flowchart of proposed fault diagnosis method for rolling bearing is given in Figure 6.

### 4.4. Analysis of Rolling Bearing Test Data

**Figure 7:**
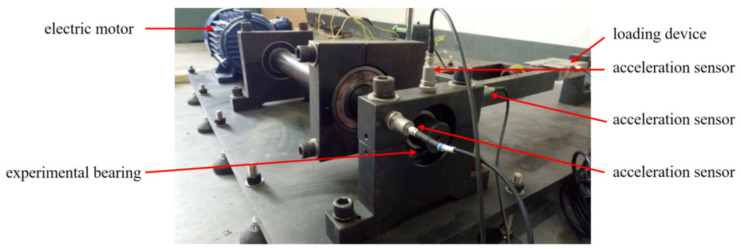
The test rig of simulated fault rolling bearing.

**Figure 8:**
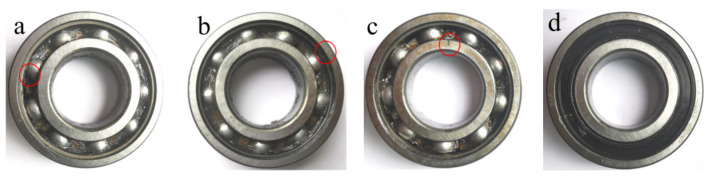
The rolling bearings with different health states for testing. (**a**) Rolling element fault; (**b**) Outer ring fault; (**c**) Inner ring fault; (**d**) Normal.

In this subsection, the test data of faulty bearings are considered as an example to test the practicability of the proposed fault diagnosis method. The test data were obtained by our group at Anhui University of Technology. The test rig is shown in Figure 7 and the different states of rolling bearings for testing are shown in Figure 8. The tested bearing model is 6206-2RS1 SKF and a single point of fault is seeded by electrical discharge machining technology. The sampling frequency is 10, 240 Hz and the specific experimental data are given in Table 1.

**Figure 9:**
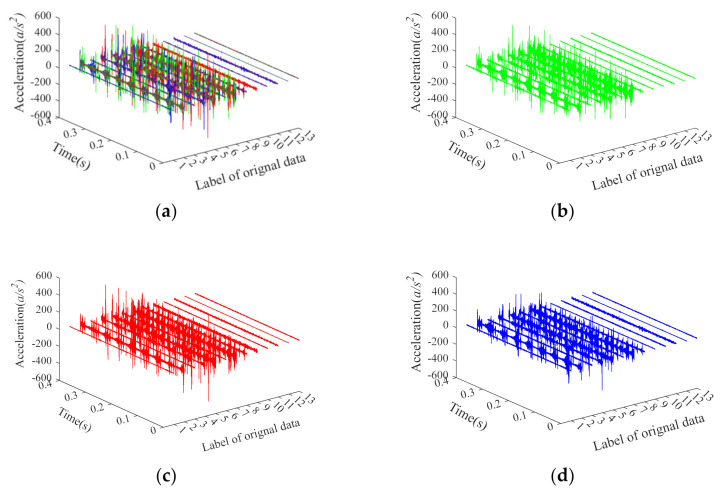
The time-domain waveform of three-channel (X, Y, Z) signals under 13 categories. (**a**) The three-channel X, Y, Z; (**b**) The single-channel X; (**c**) The single-channel Y; (**d**) The single-channel Z.

There are some issues about Table 1 that need to be illustrated. First, in each category 50 samples are selected, and thus a total of 650 samples are obtained as fault features for classification. Second, 20 samples in each category are randomly selected as training samples and the remaining 30 samples are considered as testing samples. Third, by changing the applied load, the motor speed, and the type of fault, vibration data of 13 different states are obtained. BA1, BA2, BA3, OR1, OR2, OR3, OR4, IR1, IR2, IR3, IR4, Norm1, and Norm2 are labeled as categories 1 to 13 for classification, respectively, where BA stands for the rolling element fault, OR represents the outer ring fault, IR means the inner ring fault, and Normal is the normal rolling bearing. Finally, each sample with 4096 data points is selected, and the time-domain waveform of the three-channel (X, Y, Z) signals are presented in Figure 9, where X, Y, and Z represents the sampling channels, respectively.

**Figure 10:**
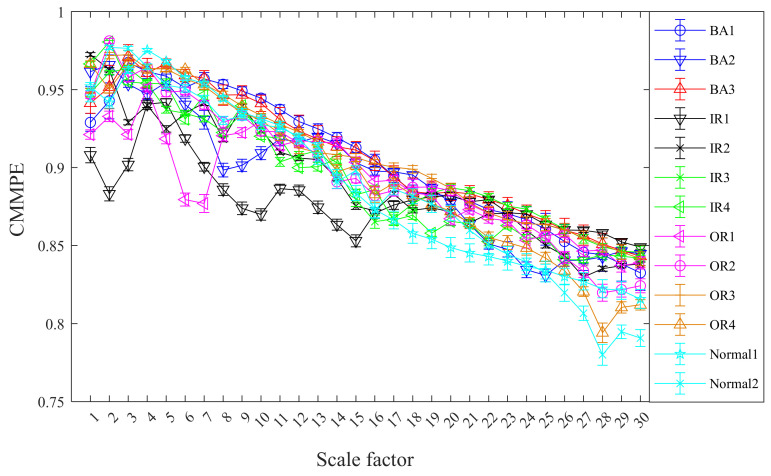
The mean standard deviation curves of CMMPE under different scale factors before LS.

**Figure 11:**
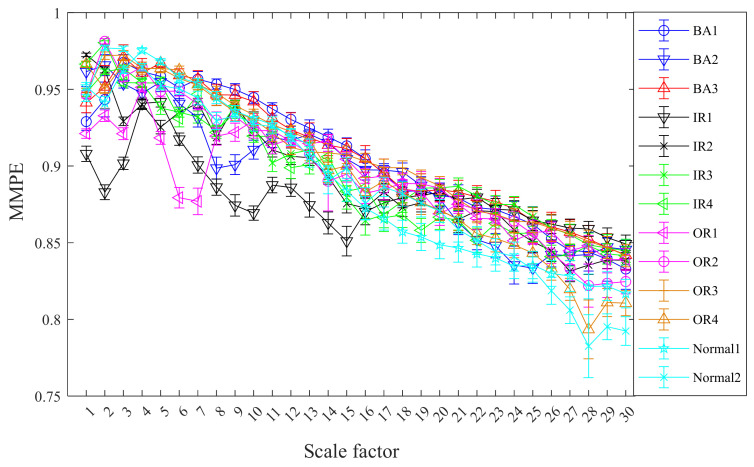
The mean standard deviation curves of MMPE under different scale factors before LS.

Without loss of generality, the mean standard deviation of CMMPE for 13 states vibration signals is computed with 30 scale factors and the results are exhibited in Figure 10. The mean standard deviation of MMPE under 13 states vibration signals are calculated as well for comparison, and the results are presented in Figure 11. We can see from Figure 10 and Figure 11 that the feature values under the 13 different categories are different and decreased gradually with the increasing of scale factor, which is consistent with the actual situation. In addition, the mean standard deviation of CMMPE under different scale factors is generally smaller than that of MMPE, which shows that CMMPE is much more stable than MMPE in fault feature extraction.

**Figure 12:**
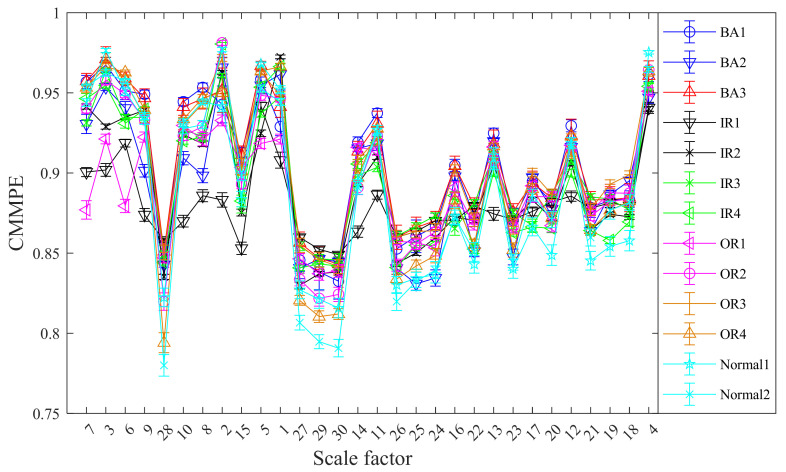
The mean standard deviation curves of CMMPE under different scale factors after LS.

**Figure 13:**
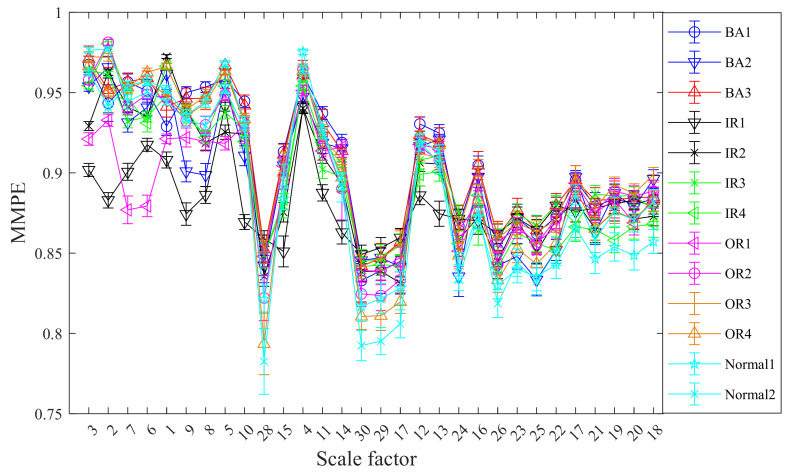
The mean standard deviation curves of MMPE under different scale factors rearranged by LS.

Next, the LS method is used to reorder the raw features. The reordered CMMPE values based on the LS scores from low to high and the mean standard deviation curves after LS are shown in Figure 12. The order of MMPE rearranged by LS scores from low to high and the mean standard deviation curves are shown in Figure 13. We can find from Figure 12 and Figure 13 that the features reordered by LS show a regular fluctuation. The features with a large difference are ranked ahead and the features with a small difference are ranked back, which indicated that the features with relatively good performance have been effectively sorted by LS scores. In this paper, for comparison, the first 1, 2, 3, …, 20 sensitive features rearranged by LS will be selected as sensitive features for fault mode identification.

**Figure 14:**
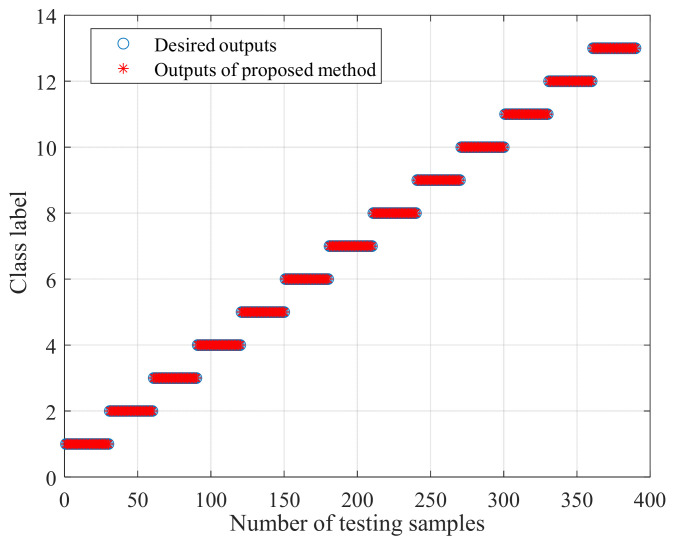
The fault identification result of proposed method under first eight sensitive features rearranged by LS.

**Figure 15:**
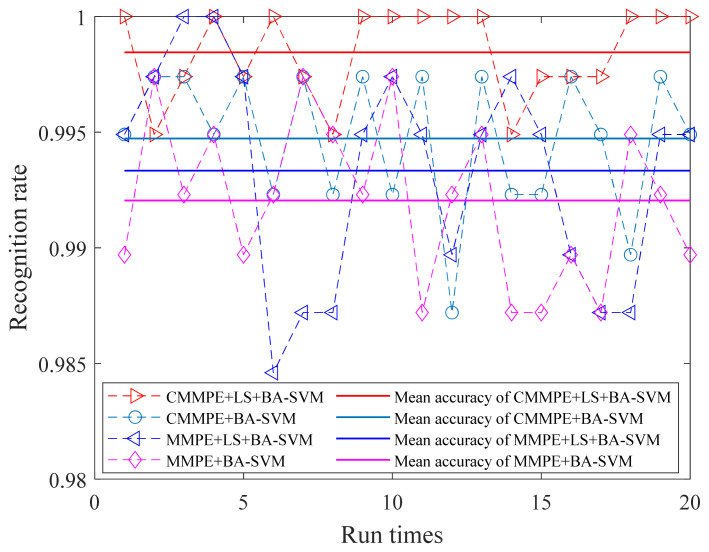
The recognition rate and the corresponding mean accuracy of different methods under 20 experiments.

Then, the first eight sensitive features of CMMPE and MMPE that were reordered by LS (namely 7, 3, 6, 9, 28, 10, 8, 2 and 3, 2, 7, 6, 1, 9, 8, 5, respectively) and the original first eight features without LS are selected for calculation. The fault identification results of the CMMPE, LS, and BA-SVM methods under the first eight sensitive features is shown in the Figure 14, from which it can be found that all the fault types can be accurately classified by the proposed method. In order to avoid false positive or false negative results, 20 experiments were conducted by the four methods under the uniform conditions, and the results are shown in the Figure 15, from which it can be seen that the proposed intelligent diagnosis method achieves better stability and the highest mean recognition rate. The mean accuracy of recognition rate of the LS based methods is higher than the corresponding methods without LS, which shows that the sensitive features can be selected by the LS method effectively. Moreover, the CMMPE based intelligent fault diagnosis methods are better than the corresponding MMPE based methods, further indicating that CMMPE is better than the MMPE method in feature extraction.

**Figure 16:**
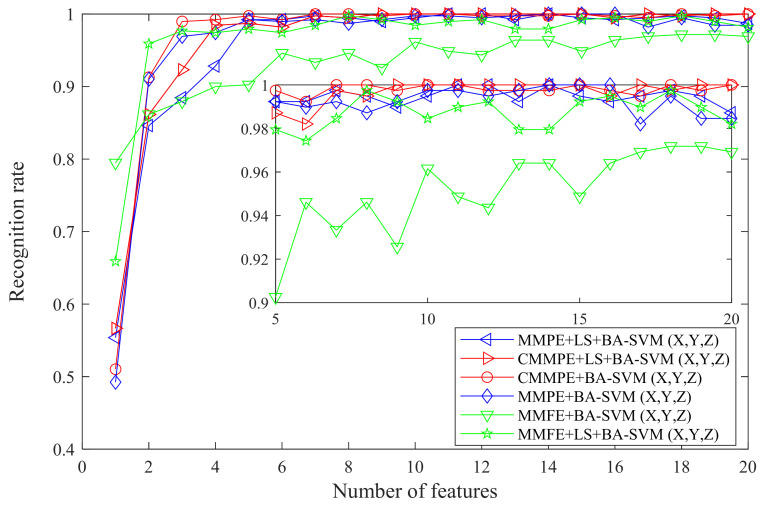
The recognition rates of testing samples by CMMPE, MMPE, and MMFE methods without LS and rearranged by LS.

For further comparison, the CMMPE, MMPE, and MMFE feature sets without LS and rearranged by LS were put into the BA-SVM based multi-classifier for comparison, and the outputs are given in Table 2. The comparison of the six methods is drawn in Figure 16, from which it can be seen that when using less than five features for calculation, the recognition rates of different methods varied greatly, due to the recognition that accuracy cannot be guaranteed by insufficient fault features. According to the comparison between CMMPE, MMPE, and MMFE based methods, it can be seen that the relatively stable recognition rate obtained by the proposed fault diagnosis method is greater than 99%, while there is a large fluctuation in the outputs of MMPE (or MMFE), LS, and BA-SVM based fault diagnosis methods. The above analysis indicates that CMMPE has a better feature extraction capability and stability than the MMPE and MMFE methods.

**Figure 17:**
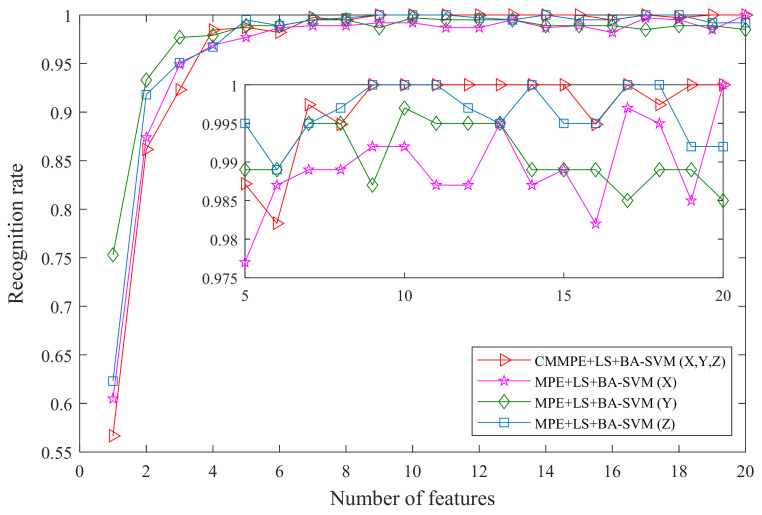
The recognition rate of different fault diagnosis methods.

Finally, to further illustrate the usability of the proposed fault diagnosis approach in handling the multi-channel data, the MPE based fault diagnosis method is used to handle the single-channel data from X, Y, and Z for comparison. The outputs of the different fault diagnosis methods are given in Table 3, and the comparison result is shown Figure 17. From this it can be seen that when more than eight features are used for calculation, the proposed fault diagnosis method maintains a higher recognition rate than that of the MPE based fault diagnosis method, whichever channel data are selected for computation, although relatively good recognition rates are obtained in the MPE based fault diagnosis method when considering Z channel data. Similarly, the MPE based fault diagnosis method gets low recognition rates when X or Y channel data are used for calculation. The results reveal that the proposed fault diagnosis method for multichannel data is superior to the single-channel signal-based method.

## 5. Conclusions

An improved nonlinear dynamic method named CMMPE is proposed in this paper to measure the complexity and dynamic mutation of multi-channel time series, which can effectively address the insufficient coarse-grained process in MMPE. The simulated signals are used to verify the validity and stability of CMMPE by comparing it with the MMPE method. An intelligent fault diagnosis method for rolling bearing is then put forward based on the CMMPE, LS, and BA-SVM methods. The test data of fault rolling bearings are applied to the proposed fault diagnosis method; the results indicate that the proposed fault diagnosis method gains outstanding stability and is better than the MMPE, MMFE, and MPE based fault diagnosis methods in feature extraction and fault identification. In addition, the difficulty of signal channel selection can be solved by the proposed fault diagnosis method, and multi-channel data can obtain a higher fault recognition rate than single-channel data. However, the parameters involved in CMMPE are still relaying the users’ knowledge, and a further study for adaptive parameter setting is needed.

## Figures and Tables

**Table 1:** The explanation of rolling bearing test data.

Fault Categories	Fault Degree (mm)	Load (kN)	Rotation Speed (r/min)	Number of Training Samples	Number of Testing Samples	Type Labels
BA1	0.2	5	900	20	30	1
BA2	0.4	5	900	20	30	2
BA3	0.2	0	1500	20	30	3
OR1	0.2	5	900	20	30	4
OR2	0.3	5	900	20	30	5
OR3	0.2	0	1500	20	30	6
OR4	0.3	0	1500	20	30	7
IR1	0.3	5	900	20	30	8
IR2	0.4	5	900	20	30	9
IR3	0.3	0	1500	20	30	10
IR4	0.4	0	1500	20	30	11
Normal1	0	5	900	20	30	12
Normal2	0	0	1500	20	30	13

**Table 2:** The recognition rate of different methods (%).

**Number of Features Used**	**1**	**2**	**3**	**4**	**5**	**6**	**7**	**8**	**9**	**10**
CMMPE+LS+BA-SVM	**56.67**	**86.15**	**92.31**	**98.46**	**98.72**	**98.21**	**99.74**	**99.49**	**100**	**100**
CMMPE+BA-SVM	51.03	91.28	98.97	99.23	99.74	99.23	100	100	99.74	100
MMPE+LS+BA-SVM	55.38	84.62	88.46	92.82	99.23	99.23	99.74	99.49	98.97	99.49
MMPE+BA-SVM	49.23	91.03	96.92	97.44	99.23	98.97	99.23	98.72	99.23	99.74
MMFE+LS+BA-SVM	65.90	95.90	97.69	97.44	97.95	97.44	98.46	99.74	99.23	98.46
MMFE+BA-SVM	79.49	86.15	87.95	90.00	90.26	94.62	93.33	94.62	92.56	96.15
	**11**	**12**	**13**	**14**	**15**	**16**	**17**	**18**	**19**	**20**
CMMPE+LS+BA-SVM	**100**	**100**	**100**	**100**	**100**	**99.49**	**100**	**99.74**	**100**	**100**
CMMPE+BA-SVM	100	99.74	99.74	99.74	100	99.74	99.49	100	99.74	100
MMPE+LS+BA-SVM	100	99.74	99.74	99.74	100	99.74	99.49	100	99.74	100
MMPE+BA-SVM	100	100	99.23	100	99.49	99.63	99.49	99.74	99.49	98.72
MMFE+LS+BA-SVM	98.97	99.23	97.95	97.95	99.23	99.49	98.97	99.74	98.97	98.21
MMFE+BA-SVM	94.87	94.36	96.41	96.41	94.87	96.41	96.92	97.18	97.18	96.92

**Table 3:** The recognition rate of different methods (%).

**Number of Features Used**	**1**	**2**	**3**	**4**	**5**	**6**	**7**	**8**	**9**	**10**
MPE+LS+BA-SVM (X)	62.82	83.85	94.36	97.69	97.44	96.92	98.97	99.74	98.72	99.49
MPE+LS+BA-SVM (Y)	73.85	97.95	98.72	99.74	99.23	99.49	99.74	98.97	99.49	99.49
MPE+LS+BA-SVM (Z)	63.08	92.05	96.41	97.18	99.23	99.49	99.23	99.74	99.49	100
	**11**	**12**	**13**	**14**	**15**	**16**	**17**	**18**	**19**	**20**
MPE+LS+BA-SVM (X)	99.23	98.72	100	98.72	98.97	99.49	98.21	99.23	99.74	98.97
MPE+LS+BA-SVM (Y)	99.74	98.97	99.23	99.74	99.23	98.97	98.72	99.23	99.23	99.23
MPE+LS+BA-SVM (Z)	99.74	99.74	99.49	99.74	99.74	99.74	99.74	100	99.74	98.72

## Data Availability

Not applicable.
